# A database of multi-principal element alloy phase-specific mechanical properties measured with nano-indentation

**DOI:** 10.1016/j.dib.2024.110719

**Published:** 2024-07-06

**Authors:** Edwin Gienger, Justin Rokisky, Denise Yin, Elizabeth A. Pogue, Bianca Piloseno

**Affiliations:** Research and Exploratory Development Department, The Johns Hopkins University Applied Physics Laboratory (JHU/APL), Laurel, MD 20723, USA

**Keywords:** Materials science, High entropy alloys, Mechanical properties, MPEAs

## Abstract

Multi-principal element alloys (MPEAs) have been the focus of study and computationally-guided design for two reasons. MPEAs have shown high strengths and, the vast potential compositional space is more efficiently navigated with machine learning. In this article, we present data from 7385 indentation tests performed on 19 different MPEAs. Samples were arc melted, a thermodynamically complex process forming many distinct phases within a sample. The database was generated by performing hundreds of nanoindentation tests on a given sample and registering the location of those indents with local phase compositions measured with energy dispersive spectroscopy (EDS). The database contains the phases formed in the MPEA, the composition at the location of each indent, and the associated hardness (HV) and modulus for each indent. This data allows researchers targeting data-driven design of high strength systems to extract meaningful correlations between alloying composition, the resulting phases, and mechanical properties for future study.

## Graphical abstract

**Figure:**
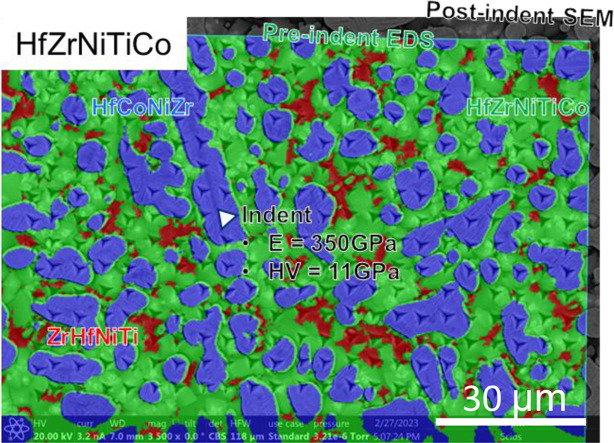

Specifications Table

**Table:** 

Subject	Materials Science: Metals and Alloys
Specific subject area	Multi-principal element alloys (MPEAs)
Type of data	Table (.csv) Analyzed and post-processed
Data collection	Nineteen alloys were produced via arc melting from raw element feedstocks. Samples were polished and imaged with scanning electron microscopy (Scios DualBeam FIB-SEM) and phases were mapped with energy dispersion spectroscopy (Oxford Ultim Max EDS detector). Following imaging, the same areas were mechanically tested with instrumented nano-indentation (KLA iMicro nanoindenter with inForce 1000 actuator) at least 280 times to a depth of at least 500 nm. Following indentation, another backscatter electron image of the same area was captured with scanning electron microscopy. A homography to align phase maps with post-indented backscatter images was discovered, and the images were aligned. This allowed for the mechanical measurements from each indent to be correlated with the phases present in the area of indentation and the composition of those phases.
Data source location	Institution: Johns Hopkins University Applied Physics Laboratory City/Town/Region: Laurel, MD Country: USA Latitude and longitude: 39.15838, −76.89942
Data accessibility	Repository name: Figshare Data identification number: 10.6084/m9.figshare.25244884 Direct URL to data: https://doi.org/10.6084/m9.figshare.25244884 Instructions for accessing these data: Navigate to the URL above using any web browser

## 1 Value of the Data

• This database can serve as a valuable resource for researchers focused on digital design for multi-principal element alloys and their development given the breadth of compositional design space and observed mechanical properties. This data can serve as a screening tool for mechanical properties of over 50 different compositions that were observed during testing.
• Since samples were arc melted and not annealed, this data can help elucidate promising phases formed in the arc melting process that were not the original compositional target.
• This data demonstrates a framework for combining multi-modal (compositional analysis and mechanical property) material measurements into a single framework.
• Lastly the data can serve as a unique training set for machine learning (ML) models used by researchers to more efficiently navigate the enormous potential compositional space presented by MPEA design.

## 2 Background

Multi-principal element alloys have been the focus of research for almost 20 years. These alloys frequently demonstrate favorable properties of several of the primary alloy elements, thus gaining traction in areas of study including mechanical light-weighting, refractory behavior, corrosion resistance, and magnetism. However, the potential composition space for these alloys is vast. There are approximately 30 elements used in combination to synthesize these materials, resulting in hundreds of thousands of potential combinations and ratios. Ideally, this design space would be navigated with data-driven design and machine learning (ML) tools, however, databases for training models to predict new materials are limited compared to datasets used to train traditional ML models. Frequently, these materials are fabricated with arc melting, a relatively high throughput technique capable of generating sufficient volumes of material for most characterization techniques. The complex thermodynamics of arc melting can result in multiple phases forming. While some researchers target certain phases with post-melt heat treatments, others use arc melting as a rapid screening technique for promising chemistries and are less concerned with the formation of phases that are not the original target. Here, we take advantage of the complex microstructures generated in arc melting, and leverage nanoindentation to measure hundreds of different data points for several phases within a given sample.

## 3 Data Description

The data is in a .csv format and hosted on the figshare platform [1]. The data set includes 104 columns of information. Each of row 3 through row 7387 contains the information from an individual nanoindentation test. The column descriptions are as follows:

• Columns A and B contain the arbitrary sample name and magnification used for analysis respectively.
• Columns C through CI contain information on each phase identified in energy dispersive spectroscopy.
  ○ Column C, N, Y, AL, BC, BN, and BY contain the name of an identified phase in a given sample. The names indicate the primary elements in the phase, but do not refer to the atomic or weight percentages of those elements. For example, sample EP3086D contains a phase named CuTiAl but the stoichiometry of that phase is actually Cu_2_TiAl.
    ■ The columns following a phase name contain the observed elements and their atomic percentage in that phase. For example, the phase in sample EP3086D named CuTiAl contains four elements, Cu, Ti, Al, and Cr, and those elements are listed in columns D, F, H, and J respectively.
    ■ The atomic percentage of each of those elements in CuTiAl are listed in columns E, G, I, and K respectively.
  ○ Not all phases contain 5 or more elements, in which case, those cells are left blank. Additionally, not all samples contain 4 or more phases, in which case, those cells are left blank.
  ○ Some phases that were aggregated in the analysis software were not assigned a name by the software. These phases are named “Unassigned”. Not all unassigned phases are the same.
• Columns CJ through CU refer to the area each phase occupies in a nano-indented area.
  ○ These values are calculated through image registration and subtraction described later.
  ○ Columns CJ, CL, CN, CO, CP, CR, and CT contain the name of a previously described phase in the sample
  ○ Columns CK, CM, CO, CQ, CS, and CU contain the percentage of area under the indent occupied by the respective phase
  ○ For example, row 34 is a test performed on sample EP3086D. The indented area is comprised of 71 % CuTiAl and 29 % CulTiAlCr
• Columns CV through CZ contain the results from each nanoindentation test
  ○ Column CV is hardness in GPa
  ○ Column CW is hardness in hardness number
  ○ Column CX is modulus in GPa
  ○ Column CY is the maximum measured load in N
  ○ Column CZ is the maximum depth from each indentation in micrometers.
• A summary of the values from this data base are described in Table 1.

**Table 1:** Features included in the database generated in this study.

Parameter	Range	Count
Hardness Value (HV)	73–3005	7385
Indentation Hardness (GPa)	0.77–31.8	7385
Modulus	40.4–360	7385
*Samples Tested*	17	
*Unique Phases Observed*	56

The process for synthesis, phase identification, and mechanical tested is listed in the experimental design, materials, and methods section below.

## 4 Experimental Design, Materials, and Methods

### 4.1 Compositional selection

Each MPEA selected for synthesis had a target five element composition. Samples were given a random name and later each indent is labeled with the specific composition. Samples EP3073A, EP3073C, and EP3078B were selected from Li et al. and Firstov et al. [2] EP3073B was selected from Hinte et al. [3] EP3073D and EP3074A-C were selected from Lee et al. [4] EP3074D, EP3076C-D, and EP3077A-D were selected from Cantor et al. [5] EP3075A-D, EP3079B-C, and EP3080B were selected from Senkov et al. [6] Of these, EP3075B accidently did not include nickel (so the mass was lower), so that sample was remade with nickel as EP303080D. EP3076A-B were selected from Fazakas et al. [7] EP3078A was selected from Zuo et al. [8] EP3078C-D were selected from Piorunek et al. [9] EP3079A was selected from Alvi et al. [10] EP3079D was an un-nitrided, bulk version of Feng et al. [11] Sample EP3080A was selected to match that of Han et al. and Soni et al. [12,13] EP3080C was from George et al. [14] EP3080D was selected from a separate George et al. publication [15]. EP3085A was selected from Knipling et al. [16] EP3085B was inspired by Knipling et al., moving down the periodic table [16]. Samples EP3085C-D, EP3086A-D, EP3087A-B, EP3088A, and EP3089D were chosen such that the electron count equaled 7, suggesting that an instability between BCC and FCC forms might be present. Samples EP3087C-D, EP3088B-D, EP3089A-C, were representative of MPEAs discussed in Y. Qiu et al. [17]. While all of the samples were fabricated, only a selection of them (17) were fully characterized due to project constrains. Those 17 samples are detailed in Table 2 below and included in the figshare repository.

**Table 2:** Characterized arc melted samples included in database.

Sample ID	Target composition	Sample ID	Target composition
EP3076C	VFeCoNiCu	EP3087A	AlTiMnNiCu
EP3076D	MnFeCoNiCu	EP3087B	Al2TiFeNi2Cu
EP3077A	VFeCoNiCu	EP3088A	AlCr2Fe3Mn2Ni
EP3077B	MnFeCoNiCu	EP3088B	AlCoCrFeNi
EP3077C	FeCoNiCu	EP3088C	CoCrFeNiCu
EP3077D	Mn5Fe6CiBu5Cu3	EP3088D	AlCoFeNiCr
EP3080A	VNbMoTaW	EP3089A	CoCrFeNiTi
EP3086C	AlVCrNiCu	EP3089B	CoCrFeNiMo
EP3086D	AlTiCrCu2		

### 4.2 Arc melting

Elemental foils were weighed and placed in arc melting sample hearths. Elemental purity is included in the associated figshare. Samples were arc melted using a MRF SA-200 arc melt furnace with currents up to 120 A under argon. Samples were melted at 75 A. Each sample was melted 2 times, flipped, and then melted 1 or 2 more times to encourage homogeneity. To account for volatilization, samples containing chromium or aluminum were made with ∼10 wt% additional of those components. Samples were not heat treated after melting. The goal of this effort is to provide phase specific mechanical information, and sample annealing would eliminate the complex microstructures in as-melted samples we were trying to characterize.

### 4.3 Sample preparation

The arc melted samples were sectioned and cold-mounted in epoxy, with up to 10 samples in one mount for expediency of preparation and characterization. This enables programming of more samples at a time for automated nanoindentation testing. The samples were prepared under standard metallographic procedures down to 1200 grit, followed by a 1 µm alumina polish and finished with 20 nm colloidal silica. While the 10 or so samples prepared within each mount do have different compositions, and thus different polishing rates, each mount was polished such that the hardest samples in the mount were sufficiently polished by the end.

### 4.4 Scanning electron microscopy/energy-dispersive spectroscopy

Location markers were placed on the surface of polished coupons by using sharp tweezers. Backscatter electron images were taken at specified distances from the end of the scratches using a dedicated detector at 20 kV accelerating voltage and 3.2 nA current. A pre-defined set of elements was entered for each alloy based on the list of elements known to be included in the synthesis. For each alloy, one image was taken with a 350 µm × 240 µm field of view (600X magnification) and another in a different area with a 120 µm × 82.4 µm field of view (1750X magnification) at high resolution (384 dpi).

In the same region of the backscatter image, an EDS map was acquired using an FEI (now Thermo Fisher Scientific) Scios DualBeam FIB-SEM equipped with an Oxford Ultim Max EDS detector. Maps were acquired with nominal dead times of 30 % and an acquisition time set to a fixed duration of 5 min (approximately 25 frames) for the 1750X magnification maps and 10 min (approximately 50 frames) for the 600X magnification maps. The map resolution was set to 512 × 352 pixels, and a dwell time per pixel of 50 µs was used.

In the Analyze Phases module, the AutoPhaseMap tool was used to convert maps into phase maps in order to see the constituent elements of each phase and how the phases are distributed over the surveyed area. The AutoPhaseMap tool is designed to automatically separate different chemical compositions in the specimen based on the spectrum information. It should be emphasized here that the “phases” identified by this tool in the software are based purely on chemistry and do not consider crystal structure.

The phase data were processed in the software by setting the boundary tolerance and grouping level (typically a different value for each map). The boundary tolerance controls the behavior at the boundaries of each phase. If the tolerance is low, each pixel in the phase represents a pure spectrum. If a pixel has contributions from several phases, it cannot be identified and appears black. If the boundary tolerance is high, all pixels are placed in the phase that fits them most closely. The spectra for each phase show small contributions from adjacent phases. The grouping level defines the extent to which similar phases are combined, e.g., to create a smaller, more manageable number of phases. If the grouping level is low, a large number of phases is displayed; phases may occupy small areas and might indicate trace compounds. Based on the aforementioned description of the post-processing workflow, it is clear that some level of user discretion is required as the phase constituents are manipulated based on and referenced back to contrast levels in the backscatter images. One would expect this method to be less sensitive when distinguishing phases that differ only slightly in composition from nearby phases. It would not distinguish regions of the same composition but different crystal structures.

After hardness testing (described below), samples were returned to the SEM for final imaging with the backscatter electron detector (using the same settings as described above) in order to register the location of indents in the nanoindentation arrays with respect to the microstructure.

### 4.5 Hardness testing

After areas of interest were mapped with EDS, instrumented indentation using a KLA iMicro nanoindenter (inForce 1000 actuator with force actuation up to 1 N) was used to apply an array of indents under load control to the imaged areas. For images taken at 600X magnification, a 14 × 20 array of indents (14 across and 20 down) was performed with 20-µm spacing and 2000 nm depth, and for images taken at 1750X magnification, a 15 × 23 array of indents was performed with 5-µm spacing and 500 nm depth, totaling 280 and 345 indents per array, respectively. Efforts were made using the 10X optical objective in the nanoindenter to align the indentation array with the field of view used to collect EDS data as best as possible, although slight deviations were common due to errors associated to repositioning the samples in the sample holder for nanoindentation. This was acceptable due to the corrections that can be applied through database generation algorithms. For instance, when only the top or bottom half of the indentation array overlayed the EDS field of view, the indents that were not located in the EDS field of view were not considered in the database as they did not have corresponding EDS phase data. Indents that were located in the EDS field of view were appropriately linked to the EDS phase data.

### 4.6 Image registration and database generation

The dataset was created with a web browser based graphical interface which allowed each stage of the data processing pipeline to be visually validated for accuracy. The pipeline consisted of five stages:

1. Uploading the backscatter image of the sample, the associated EDS maps, and inputting the collected data about the compounds present in the EDS maps. These EDS maps were transformed into binary images and overlaid on the backscatter image. Each phase was given a unique color. All EDS maps were fused into a single image.
2. Uploading the image of the sample after indentation, as well as the collected data for each indent. After the upload was complete, bounding triangles were marked and an indentation mask was created.
3. Discovering a homography to align the aggregate EDS image into the space of the post-nanoindented image. Pairs of points representing the same location of the sample in each image were identified and then used to compute a homography. A first attempt to find valid matches was performed algorithmically by extracting scale invariant features (SIFT)[18] features and performing a brute force match on them. If this did not yield valid results, the user provided matching points.
4. Validating the accuracy of the homography previously computed. This was done by applying the homography to the EDS image and overlaying it on top of the post-nanoindented image. The output was visually inspected to ensure that features aligned. The user also had the option to select a point in the EDS image and ensure it was mapped (via the computed homography) to the expected location in the post-indentation image.
5. Applying the bounding triangles as a mask to the homographically transformed EDS image to remove all non-indented areas of the EDS image. For each bounding triangle, we computed the ratio of each color inside the triangle which allowed us to compute the percent of each composition present in the triangle as each compound was mapped to a specific color. The indentation values for each indent were assigned to the corresponding triangle.

### 4.7 Data validation

**Fig. 1:**
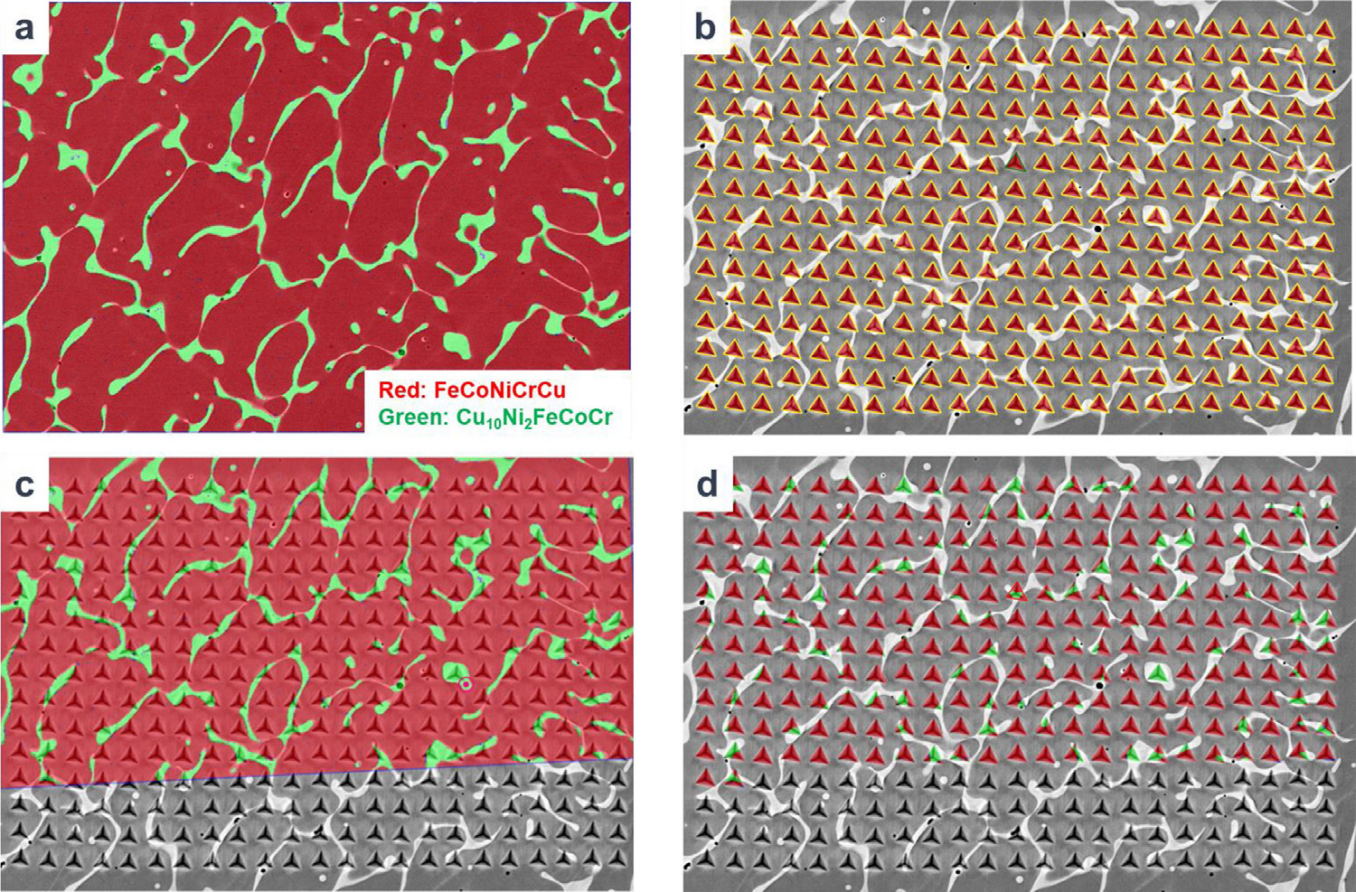
Workflow for data analysis. (a) Registration of the binary EDS maps [step 1], (b) confirmation of triangle maps on each indent [step 2], (c) registration of EDS maps with post-indentation images [steps 3 and 4] and (d) validation of EDS maps overlaid on each indent [step 5].

Data from all samples regardless of hardness were included in the database. Several invalid, individual indents were removed from the dataset for one of two reasons. Some indentation tests reported negative values for hardness or modulus. This is likely attributed to an invalid indent that did not properly engage the surface due to as-melted cracking or porosity. Other individual indents were removed because their values were in excess of 10,000 HV in the same phase as indents that measured a hardness below 1000 HV. This would make them some of the hardest materials ever reported, and were also likely bad tests for reasons mentioned previously (Fig. 1).

**Fig. 2:**
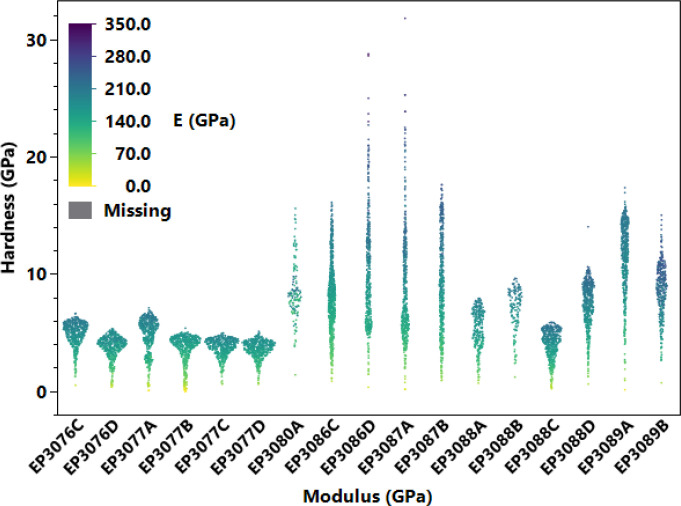
Reported mechanical properties (modulus and hardness) of measured samples.

However, all validate indents are included regardless of whether or not the samples were high hardness. This apparent in the range of results where hardness values range from 73 to 3000 HV. A summary of the hardness and modulus for each sample is presented in Fig. 2.

## Limitations

### Phase identification

It is clear that some level of user discretion is required as the definition of a single phase and phase constituents are manipulated based on and referenced back to contrast levels in the backscatter images. One would expect this method to be less sensitive when distinguishing phases that differ only slightly in composition from nearby phases. It would not distinguish phases of the same composition but different crystal structures. Kinetically achieved composition gradients within a single solid-solution phase region may also be challenging to classify correctly using this method.

### Mechanical properties

Each indentation is registered to the surface phases observed in SEM/EDS. However, the depth of each phase in unknown. Therefore, it is possible that indents are engaging other phase compositions as well in addition to the surface phase that is reported.

Not all 7000+ mechanical tests were individually verified and therefore may contain some outliers.

## Ethics Statement

The authors have read and follow the ethical requirements for publication in Data in Brief and confirm that the current work does not involve human subjects, animal experiments, or any data collected from social media platforms.

## CRediT authorship contribution statement

**Edwin Gienger:** Conceptualization, Methodology, Validation. **Justin Rokisky:** Methodology, Software, Data curation, Visualization. **Denise Yin:** Methodology, Formal analysis. **Elizabeth A. Pogue:** Methodology, Formal analysis. **Bianca Piloseno:** Methodology, Formal analysis.

## Data Availability

A database of multi-principal element alloy phase-specific mechanical properties measured with nano-indentation (Original data) (Figshare).
